# Health data from diaries used in low-income communities, north India

**DOI:** 10.2471/BLT.20.264325

**Published:** 2021-04-01

**Authors:** Neeta Kumar, Tulsi Adhikari, Jiten Kh Singh, Nidhi Tiwari, Anita S Acharya

**Affiliations:** a Indian Council of Medical Research, Ansari Nagar, New Delhi 110029, India.; b National Institute of Medical Statistics, New Delhi, India.; c Lady Hardinge Medical College, New Delhi, India.

## Abstract

**Objective:**

To determine the acceptability of keeping a self-written health diary among members of low-income communities, with the aim of generating needed health data.

**Methods:**

We identified three different types of impoverished communities (tribal, inner-city slum and rural) in north India, and conducted a baseline survey to establish the sociodemographic properties of the members of 595 (tribal), 446 (slum) and 51 (rural) households. We designed health diaries with a single page to fill in per month, each with a carbon duplicate, and distributed diaries to willing participants. Health volunteers visited households each month to assist with diary completion and to collect duplicate pages for a period of one year. We compared the frequency of illnesses reported in health diaries with baseline survey data.

**Findings:**

A total of 4881 diary users (tribal: 2205; slum: 2185; rural: 491) participated in our project. In terms of acceptability, 49.6% (1093/2205), 64.7% (1413/2185) and 79.0% (388/491) at the tribal, slum and rural sites, respectively, expressed satisfaction with the scheme and a willingness to continue. In the tribal and slum areas, we observed increased reporting of illnesses from health diaries when compared with baseline data. We observed that influenza-like illnesses were reported with the highest frequency of 58.9% (2972/5044) at the tribal site.

**Conclusion:**

We observed high levels of acceptability and participation among the communities. From our initial field studies, we have observed the benefits to both our study participants (timely preventive education and referrals) and to service providers (obtaining health data to allow improved planning).

## Résumé

**Objectif:**

Déterminer si les membres des communautés à faible revenu sont prêts à accepter de tenir eux-mêmes un carnet de santé afin de générer les données requises en la matière.

**Méthodes:**

Nous avons identifié trois différents types de communautés défavorisées (communautés tribales, bidonvilles en milieu urbain et communautés rurales) en Inde du Nord. Nous avons mené une enquête de référence afin de définir les caractéristiques sociodémographiques des membres de 595 ménages parmi les communautés tribales, 446 dans les bidonvilles et 51 parmi les communautés rurales. Nous avons conçu des carnets de santé comportant une page à remplir par mois, chacune munie d'une copie carbone, et les avons distribués aux participants. Pendant un an, les volontaires de santé ont rendu visite aux ménages chaque mois pour les aider à compléter leur carnet et récolter les copies carbone. Nous avons comparé la fréquence des maladies mentionnées dans les carnets de santé avec les données de l'enquête de référence.

**Résultats:**

Au total, 4881 personnes ont participé à notre projet et tenu un carnet de santé (communautés tribales: 2205; bidonvilles: 2185; communautés rurales: 491). En termes d'acceptation, 49,6% (1093/2205) des membres de communautés tribales, 64,7% (1413/2185) des habitants de bidonvilles et 79,0% (388/491) des membres de communautés rurales ont exprimé leur satisfaction vis-à-vis du système et sont prêts à continuer. Dans les deux premières communautés, nous avons remarqué qu'un plus grand nombre de maladies étaient signalées dans les carnets de santé que dans les données de référence. Nous avons également constaté que c'est dans les communautés tribales que les syndromes grippaux ont été les plus fréquemment rapportés, avec 58,9% (2972/5044).

**Conclusion:**

Nous avons observé un niveau élevé d'acceptation et de participation au sein des communautés. En examinant nos études initiales sur le terrain, nous avons pu voir les avantages de cette méthode pour nos participants (éducation préventive et renvoi vers un spécialiste dans un délai convenable) et pour les prestataires de services (obtention de données sur la santé permettant d'améliorer la planification).

## Resumen

**Objetivo:**

Determinar la aceptabilidad de llevar un diario de salud escrito por uno mismo entre los miembros de las comunidades de bajos ingresos, con el objetivo de generar los datos de salud necesarios.

**Métodos:**

Identificamos tres tipos diferentes de comunidades empobrecidas (tribales, barrios marginales del centro de la ciudad y rurales) en el norte de la India, y realizamos una encuesta de referencia para establecer las propiedades sociodemográficas de los miembros de 595 (tribales), 446 (barrios marginales) y 51 (rurales) hogares. Se diseñaron diarios de salud con una sola página para rellenar al mes, cada uno con un duplicado en papel carbón, y se distribuyeron los diarios a los participantes dispuestos. Los voluntarios sanitarios visitaron los hogares cada mes para ayudar a rellenar el diario y recoger los duplicados durante un año. Se comparó la frecuencia de las enfermedades declaradas en los diarios de salud con los datos de la encuesta de referencia.

**Resultados:**

Un total de 4.881 usuarios de diarios (tribales: 2.205; de barrios marginales: 2.185; rurales: 491) participaron en nuestro proyecto. En cuanto a la aceptabilidad, el 49,6% (1093/2205), el 64,7% (1413/2185) y el 79,0% (388/491) de las zonas tribal, barrios marginales y rural, respectivamente, expresaron su satisfacción con el programa y su voluntad de continuar. En las zonas tribales y de barrios marginales, observamos un aumento de la notificación de enfermedades en los diarios de salud en comparación con los datos de partida. Observamos que las enfermedades similares a la gripe se notificaron con la mayor frecuencia, el 58,9% (2972/5044), en la zona tribal.

**Conclusión:**

Hemos observado altos niveles de aceptación y participación entre las comunidades. A partir de nuestros estudios iniciales sobre el terreno, hemos observado los beneficios tanto para nuestros participantes en el estudio (educación preventiva oportuna y remisiones) como para los proveedores de servicios (obtención de datos sanitarios que permiten mejorar la planificación).

## ملخص

الغرض تحديد مدى قبول الاحتفاظ بيوميات صحية مكتوبة ذاتيًا بين أفراد المجتمعات منخفضة الدخل، بهدف تجميع البيانات الصحية اللازمة.

الطريقة لقد حددنا ثلاثة أنواع مختلفة من المجتمعات الفقيرة (القبلية، والأحياء الفقيرة داخل المدن، والريفية) في شمال الهند، وأجرينا مسحًا أساسيًا لتحديد الخصائص الاجتماعية والسكانية لأعضاء الأسر من 595 (من القبائل)، و446 (من الأحياء الفقيرة)، و51 (من الأرياف). لقد قمنا بتصميم اليوميات الصحية لتكون من صفحة واحدة يتم ملئها شهريًا، وبكل منها صورة كربونية، وقمنا بتوزيع اليوميات على المشاركين الراغبين. قام متطوعون في مجال الصحة بزيارة الأسر كل شهر للمساعدة في إكمال اليوميات، وجمع الصفحات الكربونية لمدة عام واحد. وقمنا بمقارنة مدى تكرار الأمراض التي تم التبليغ عنها في اليوميات الصحية، مع بيانات المسح الأساسي.

النتائج شارك إجمالي 4881 من مستخدمي اليوميات (من القبائل: 2205؛ ومن الأحياء الفقيرة: 2185؛ ومن المناطق الريفية: 491) في مشروعنا. ومن حيث المقبولية، فإن 49.6٪ (1093/2205)، و64.7٪ (1413/2185)، و79.0٪ (388/491)، من المستخدمين في كل من المواقع القبلية والأحياء الفقيرة والريفية على التوالي، قد أعربوا عن رضاهم عن المخطط والرغبة في الاستمرار. في المناطق القبلية والأحياء الفقيرة، لاحظنا زيادة في إبلاغ اليوميات الصحية عن الأمراض، عند مقارنتها مع بيانات خط الأساس. ولاحظنا أنه تم الإبلاغ عن أمراض شبيهة بالإنفلونزا بأعلى معدل تكرار قدره 58.9٪ (2972/5044) في الموقع القبلي.

الاستنتاج لقد لاحظنا مستويات عالية من القبول والمشاركة بين المجتمعات. استنادًا إلى دراساتنا الميدانية الأولية، لاحظنا الفوائد لكل من المشاركين في الدراسة (التعليم الوقائي والإحالات في الوقت المناسب)، وكذلك لمقدمي الخدمات (الحصول على البيانات الصحية للسماح بالتخطيط المحسن). ****

## 摘要

**目的:**

旨在确定低收入社区成员对于由自己坚持填写健康日记这种方式的接受度，并最终生成所需的健康数据。

**方法:**

我们发现印度北部存在三种不同类型的贫困社区（部落、内城区贫民窟和农村），于是展开基线调查，以确定各类社区家庭成员的社会人口统计学特征，其中包括部落的 595 个家庭、贫民窟的 446 个家庭以及农村的 51 个家庭。我们设计了一种每月仅需填写一页且每页均带复写页的健康日记，然后将此类日记分发给愿意参加的家庭。在连续一年内，健康志愿者每月都会上门协助他们完成日记并收集复写页。我们将健康日记中报告的患病频率与基线调查数据进行了比较。

**结果:**

共有 4881 名日记用户（部落：2205；贫民窟：2185，农村：491）参与我们这个项目。在接受度方面，部落、贫民窟和农村地区分别有 49.6% (1093/2205)、64.7% (1413/2185) 以及 79.0% (388/491) 表示对该计划很满意，并愿意继续参加。相对于基线数据，我们发现部落和贫民窟地区健康日记中报告的发病量有所增加。我们发现，部落地区报告的流感样疾病的发病率最高可达 58.9% (2972/5044)。

**结论:**

我们注意到各类社区的接受度和参与度均比较高。根据我们的初步现场研究，我们发现这种方式对调查参与者（及时进行预防教育和转诊）和服务提供者（获取健康数据，便于改善计划）都非常有利。

## Резюме

**Цель:**

Определить приемлемость ведения рукописного дневника здоровья среди членов общин с низким уровнем дохода с целью получения необходимых данных о состоянии здоровья.

**Методы:**

Авторы выделили три различных типа бедных общин (племенные общины, городские трущобы и сельские районы) на севере Индии и провели базовое исследование с целью определить социально-демографические характеристики членов 595 (племенные общины), 446 (трущобы) и 51 (сельские районы) домохозяйства. Были разработаны дневники здоровья, каждая страница которых заполняется в течение месяца и имеет дубликат, изготовленный под копирку. Дневники были розданы добровольным участникам исследования. Волонтеры-медики ежемесячно посещали домохозяйства для оказания помощи в заполнении дневников и сбора дубликатов страниц в течение одного года. Авторы сравнили частоту заболеваний, упоминаемых в медицинских дневниках, с данными базового исследования.

**Результаты:**

В общей сложности в проекте участвовали 4881 пользователь дневника (племенные общины: 2205; трущобы: 2185; сельские районы: 491). С точки зрения приемлемости 49,6% (1093/2205), 64,7% (1413/2185) и 79,0% (388/491) в племенных общинах, трущобах и сельских районах соответственно выразили удовлетворенность данной схемой работы и готовность продолжать заполнение дневников. В племенных и трущобных районах наблюдалось увеличение количества сообщений о болезнях в дневниках здоровья относительно показателей из основных данных. Авторы отметили, что на племенных территориях гриппоподобные заболевания регистрировались с самой высокой частотой — 58,9% (2972/5044).

**Вывод:**

Авторы отметили высокий уровень приемлемости и участия среди общин. Первоначальные исследования на местах демонстрируют преимущества как для участников исследования (своевременное профилактическое просвещение и направление к специалистам), так и для поставщиков услуг (получение данных о состоянии здоровья для улучшения планирования).

## Introduction

The third sustainable development goal (SDG) aims to ensure healthy lives and promote well-being for all at all ages. The 2030 SDG targets include reducing mortality through education and prevention, improving accessibility to health-care services for all, and strengthening the capacity of countries for early warning and management of global health risks.^1^ However, despite increased funding for health care in India,^2^^,^^3^ the complex and diverse socioeconomic and demographic situation in this country means that many communities cannot access adequate health care. Achieving health equity requires widely encompassing and up-to-date data on the health conditions of a population; without data, policy-makers cannot determine the effect of interventions on public health and inequalities.^2^^–^^4^ However, inadequate funding and lower priority, particularly in low- and middle-income countries, mean that such data are not always available. 

Currently, front-line health workers in India record health data in their register or on the digital platform given to them for data entry. However, this means that (i) information can be recorded without actually contacting and visiting the patient; and (ii) data are only compiled on a specific list of notifiable diseases,^5^ mostly from hospital data, and exclude transient symptoms of common influenza-like illnesses.^6^ To bridge this data gap, community participation in the generation of health data has been identified as a core value of authentic health impact assessment.^4^ Self-sustaining data collection mechanisms and accompanying two-way channels of communication between health workers and the community have been emphasized in the literature.^4^

To generate needed and more complete health data for impoverished communities (tribal, inner-city slum and rural) in India, we investigated the possibility of community members keeping a regular health diary. We aimed to determine the acceptability of such an intervention among members of these communities, as well as its feasibility in terms of helping with the periodic updating of the diary where necessary and retrieving recorded data.

## Methods

### Study design

We identified three impoverished areas of different types for our investigation, which took place over three different 3-year periods: the tribal area of Arunachal Pradesh (2015–2018), an inner-city slum in Delhi (2016–2019) and the rural area of Hardoi (2012–2015). We originally requested that relevant state secretaries assist us in obtaining 2500 potential participants at each study site by identifying 500 households within their area; the nonsimultaneous study periods were a result of the variable dates of response and approval. We changed our target study population to only 500 people for the rural study (Hardoi) as it was the first site, allowing us to test our system, and visited 595 households in the tribal study (Arunachal Pradesh) and 446 households in the inner-city slum (Delhi) to achieve our target study populations (Table 1).

**Table 1:** Properties of field sites and community participation in study to determine acceptability of self-written health diary, north India, 2012–2019

Property	Location			
	Arunachal Pradesh (tribal)	Delhi (slum)		Hardoi (rural)
Study period	2015–2018	2016–2019		2012–2015
No. potential participants approached	2 500	2 500		500
No. households	595	446		51
Average no. household members	4.2	5.6		9.8
No. distributed diaries	2 205	2 185		491
No. health diary pages retrieved during monthly visits	13 776	17 142		4 721
No. who expressed satisfaction and willingness to continue, regardless of no. diary pages completed	1 093	1 413		388
No. diaries retrieved at study end	1 146	1 442		392
No. diaries lost or damaged	1 059	743		99

**Fig. 1:**
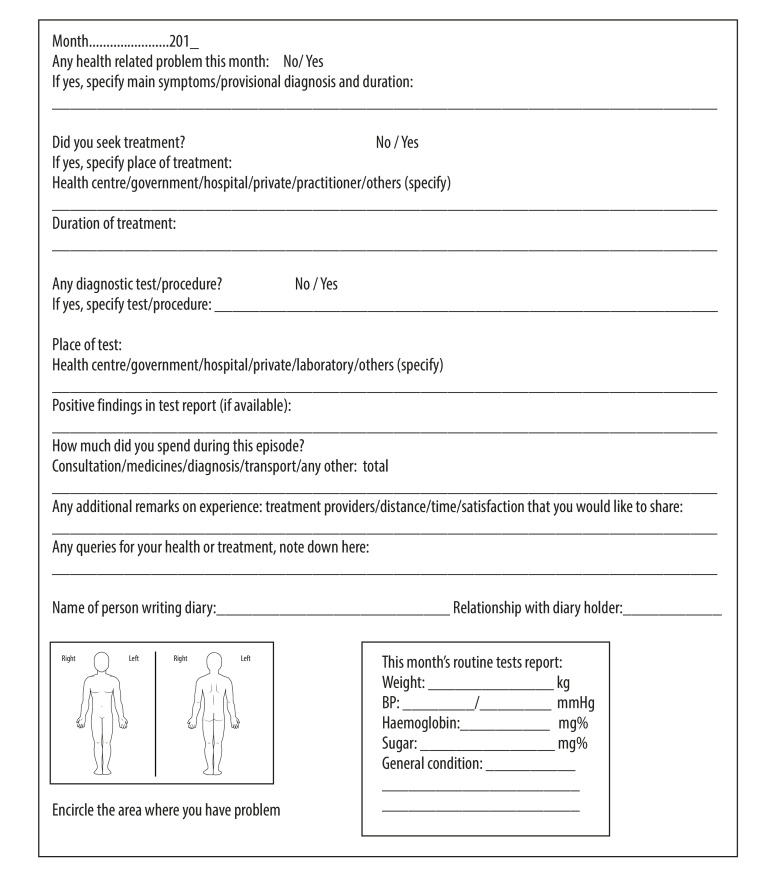
Format of monthly health diary page used in a study to determine diary acceptability, north India, 2012–2019

After wide discussion with public health experts and social scientists, we designed the health diary to have a single page with a carbon duplicate to be filled in for each month (Fig. 1). Space to record the participant’s unique identification number was provided on each page. We selected an update frequency of one month on the basis of an acceptable period for recall;^7^ although shorter update periods would reduce recall bias, they may also have resulted in reduced compliance.^8^

### First year

During the first year of each 3-year study period, we obtained the assistance of a health administrator at each site to manage all workers recruited to our project; this was the chief medical officer of the district hospital at the tribal and rural sites, and a member of the Department of Social and Preventive Medicine at Lady Hardinge Medical College for the inner-city slum. Each site administrator appointed a team of four staff (medical officer, data entry operator, computer programmer and social worker) who received a salary from a task force study fund provided by the Indian Council of Medical Research. We provided each team with a laptop, screening equipment for measurement of height, weight and blood pressure, glucose and haemoglobin, and health diaries for distribution. Although the households within each site were randomly chosen, each study team ensured their sample was representative by checking that the households spanned both central and peripheral areas of their study site.

We designed a baseline survey for demographic profiling that was conducted by each site team on a door-to-door basis. We trained all members of the teams involved in the survey with the assistance of a coordinating team of site investigators of the Indian Council of Medical Research. As well as basic demographic information on education level, employment and income, we sought information on the usual methods and frequency of health-care seeking of the community members, usual health-care expenses incurred and attitude towards sharing information recorded in a personal health diary. We conducted all baseline interviews with the designated main guardian of each family, who agreed to keep a health diary and provide consent for all members of the household younger than 18 years. 

### Second year

We distributed health diaries to participants and provided guidance in completing each month’s page during brief and informal doorstep exchanges. We instructed our initial field workers, Accredited Social Health Activists who are recruited and remunerated by the Government of India, on diary page assistance and collection, and on basic health care. However, 3 months into our first diary collection period, we learned that because the focus of the health activists is normally maternal and childcare, they were failing to visit their assigned households at the required times (citing lack of time, resources and incentives). We therefore began again, but this time using study staff or community health volunteers that we invited to come forward during local community meetings. We provided our volunteers with basic health-care training and preventive health education, making use of the health activist training manual, and on the collection of duplicate diary pages. 

Until the health volunteers took over, our study staff visited households at the end of each month to assist with diary completion (of 4881 participants, 772 were known to be illiterate and 576 were of unknown education level, assumed to be illiterate) and to collect duplicate pages. Health volunteers recorded symptoms as reported by the diary holders, and validated any actual diagnoses with available medical documents (e.g. note or receipt from doctor, caregiver or pharmacist, or packaging from prescribed medicine). Monthly visits to households also included any relevant health education and testing, as well as dispensing of basic medicines such as deworming tablets and folic acid supplements. For more complex issues, health volunteers would refer study participants to their nearest health facilities. 

### Third year

During the third year our study staff and health volunteers collected all health diaries, allowing us to calculate the number of missing or misplaced diaries. To obtain the opinion of the diary users, we designed an end-of-study survey for our survey teams to conduct by interview on a door-to-door basis. We asked study participants about any factors affecting their recording of symptoms, and sought feedback on the diary format. 

### Data analysis

We originally intended that our collected data from diary pages be uploaded to an internet application for analysis and archiving; however, the availability and speed of internet connections at all study sites made this impossible. Our study staff therefore recorded data using Excel (Microsoft, Redmond, United States of America, USA) on their provided laptops. We then coded and analysed data using frequency tables for sociodemographic variables, as well as reported symptoms and illnesses, using SPSS version 20 (IBM, Armonk, New York, USA).

### Ethics

We obtained approval for our study and diary design from the Bioethics Unit of the Indian Council of Medical Research. We then submitted our study protocol to the Institutional Ethical Committee of each site, and obtained approval as per the ethical guidelines of the Indian Council of Medical Research. We carefully followed all ethical guidelines for obtaining consent from guardians of minors (< 18 years). 

## Results

### Sociodemographic parameters 

A total of 4881 health diary users (Table 1 and Table 2) participated in our study, the largest group of which was those aged 25–39 years at all three sites. Parents and guardians provided the input data for all participants aged < 18 years. In terms of education level, we observed that the largest group of participants were educated to primary school level (124/491; 25.3%) at the rural site, and illiterate at the tribal (135 illiterate + 563 non-responders assumed to be illiterate, out of 2205 participants; 31.7%) and inner-city slum ((542+13)/2185; 25.4%) areas. The majority of families were either in the lowest (298/491; 60.7% for the rural site) or second-lowest (538/2205; 24.4% for the tribal site; 719/2185, 32.9% for the inner-city slum) income groups.

**Table 2:** Demographic profile of participants in study to determine acceptability of self-written health diaries, north India, 2012–2019

Demographic parameter	No. (%)		
	Arunachal Pradesh (tribal) (*n* = 2 205)	Delhi (slum) (*n* = 2 185)	Hardoi (rural) (*n* = 491)
**Male**	1115 (50.6)	1115 (51.0)	254 (51.7)
**Female**	1090 (49.4)	1070 (49.0)	237 (48.3)
**Age, years**			
0–4	207 (9.4)	196 (9.0)	43 (8.8)
5–9	222 (10.0)	190 (8.7)	51 (10.4)
10–17	453 (20.5)	332 (15.2)	92 (18.7)
18–24	379 (17.2)	380 (17.4)	71 (14.5)
25–39	595 (27.0)	577 (26.4)	108 (22.0)
40–64	289 (13.1)	422 (19.3)	102 (20.8)
> 65	28 (1.3)	88 (4.0)	24 (4.9)
Unknown	32 (1.5)	0 (0.0)	0 (0.0)
**Education**			
Illiterate	135 (6.1)	542 (24.8)	95 (19.3)
Primary	340 (15.4)	444 (20.3)	124 (25.3)
Middle school	345 (15.6)	421 (19.3)	92 (18.7)
High school	479 (21.8)	325 (14.9)	52 (10.6)
Intermediate	282 (12.8)	224 (10.3)	45 (9.2)
Graduate/postgraduate	53 (2.4)	205 (9.4)	63 (12.8)
Professional/honours	8 (0.4)	11 (0.5)	20 (4.1)
Unknown	563 (25.5)	13 (0.6)	0 (0.0)
**Occupation**			
Housewife	196 (8.9)	511 (23.4)	121 (24.6)
Student	744 (33.7)	664 (30.4)	171 (34.8)
Regular work	67 (3.0)	208 (9.5)	5 (1.0)
Irregular work	65 (2.9)	273 (12.5)	66 (13.4)
Business	88 (4.0)	85 (3.9)	19 (3.9)
Unemployed	36 (1.6)	280 (12.8)	15 (3.1)
Private job	50 (2.3)	107 (4.9)	15 (3.1)
Government job	201 (9.2)	2 (0.1)	20 (4.1)
Pension	2 (0.1)	55 (2.5)	9 (1.8)
Unknown	756 (34.3)	0 (0.0)	50 (10.2)
**Monthly family income (Indian rupees)^a^**			
< 6 298	313 (14.2)	459 (21.0)	298 (60.7)
6 298–10 495	538 (24.4)	719 (32.9)	47 (9.6)
10 496–15 705	467 (21.2)	419 (19.2)	35 (7.1)
15 706–20 991	417 (18.9)	225 (10.3)	29 (5.9)
> 20 991	470 (21.3)	363 (16.6)	82 (16.7)

^a^ As per Kuppuswamy scale for 2017.^9^

Note: The 2016–2017 currency rate was one United States dollar to 67 Indian rupees.^10^

### Acceptability

The proportion of community members who agreed to participate in the diary project was 98.2% (491/500) at the rural site, followed by 88.2% (2205/2500) at the tribal site and 87.4% (2185/2500) at the inner-city slum (Table 1). Of those who agreed to take part in our study, we observed the highest proportion of partly completed diaries retrieved at the end of the year of 79.8% (392/491) at the rural site; this proportion was only 52.0% (1146/2205) and 66.0% (1442/2185) at the tribal area and inner-city slum, respectively (Table 1). 

In Table 3 we demonstrate how compliance with our project varied over the year-long period of diary page collection at all three sites. Compared with both the tribal area and inner-city slum, compliance started off highest (491/491; 100.0%) and finished lowest (127/491; 25.9%) at the rural site. We noted that the most commonly reported reason for not completing the diary was migration either for work or study, prompting the suggestion that diaries and hence health data should be considered as portable.

**Table 3:** Number of health diary pages collected each month, as a percentage of number of diary users, in a study to determine diary acceptability, north India, 2012–2019

Month^a^	Arunachal Pradesh (tribal) (*n* = 2205)	Delhi (slum) (*n* = 2185)	Hardoi (rural) (*n* = 491)
1	998 (45.3)	1117 (51.1)	491 (100.0)
2	1204 (54.6)	1530 (70.0)	491 (100.0)
3	933 (42.3)	1326 (60.7)	491 (100.0)
4	866 (39.3)	1650 (75.5)	450 (91.6)
5	842 (38.2)	1578 (72.2)	435 (88.6)
6	711 (32.2)	1605 (73.5)	422 (85.9)
7	1003 (45.5)	1556 (71.2)	411 (83.7)
8	969 (43.9)	1453 (66.5)	389 (79.2)
9	1939 (87.9)	1421 (65.0)	456 (92.9)
10	1792 (81.3)	1321 (60.5)	324 (66.0)
11	1362 (61.8)	1432 (65.5)	234 (47.7)
12	1157 (52.5)	1153 (52.8)	127 (25.9)

^a^ Starting month was June, February and January in the tribal, slum and rural sites, respectively.

The most regular method of collection of the duplicate diary pages at the rural site was by the local health volunteers during routine monthly visits (153/392; 39.2%). Study staff collected the largest proportion of diary pages at the tribal site (67.0%, 768/1146) and the inner-city slum (88.3%, 1274/1442) during regular home visits (Table 4). 

**Table 4:** Survey results from participants from whom a health diary was retrieved at the end of the year in a study to determine diary acceptability, north India, 2012–2019

Study item		No. (%)		
		Arunachal Pradesh (tribal) (*n* = 1146)	Delhi (slum) (*n* = 1442)	Hardoi (rural) (*n* = 392)
**Source of assistance in completing diary**				
None		95 (8.3)	56 (3.9)	4 (1.0)
Family member, friend or relative		100 (8.7)	112 (7.8)	65 (16.6)
Study staff		950 (82.9)	1244 (86.3)	257 (65.6)
Local health volunteers		1 (0.0)	49 (3.4)	66 (16.8)
**Method of acquiring diary pages during the year^a^**				
Submitted by users		46 (4.0)	167 (11.6)	20 (5.1)
Collected by project staff during routine monthly home visit (until health volunteers took over)		768 (67.0)	1274 (88.3)	99 (25.3)
Collected by local health volunteers during routine monthly visits		252 (22.0)	86 (6.0)	153 (39.0)
Collected by project staff during additional visits made to encourage diary users^b^		126 (11.0)	80 (5.5)	139 (35.5)
**Experienced benefits of participating in health diary project^c^**		860 (75.0)	634 (44.0)	223 (56.9)
**Expectations from health diary completely fulfilled**		252 (22.0)	275 (19.1)	145 (37.0)
**Requested a single health diary for whole family**		34 (3.0)	10 (0.7)	8 (2.0)
**Requested that staff visits for diary updates include relevant testing and dispensing of medicines**		1020 (89.0)	1432 (99.3)	306 (78.1)

^a^ These numbers add up to more than 100% because some diary users experienced more than one method of page collection.

^b^ The regular community meetings held at the close-knit rural site helped to increase the number of diary pages collected.

^c^ Many diary users reported benefits of regular contact with health workers and volunteers, such as education on preventive health care, and becoming aware of other relevant and ongoing health interventions.

From the end-of-study survey, we learned that the majority of diary holders – from 306/392 (78.1%) for the rural site to 1432/1442 (99.3%) at the inner-city slum – requested that visits for duplicate page collection should include relevant testing and dispensing of medicines (Table 4). Out of all participants who agreed to keep a diary, at the end of the study we recorded user satisfaction and willingness to continue among 59.3% (2894/4881; Table 1). Participants assessed our study to be the best for increasing their awareness of other schemes; health volunteers were not only providing education on preventive health care but, during their regular visits, were also keeping participants informed of other relevant interventions (e.g. the provision of contraceptives or education on the importance of boiling water for drinking).

### Morbidity

Our data reveal that, during the year-long period of diary page collections, the reporting of illnesses increased at the tribal site from a baseline of 18.1% (399/2205) to 36.6% (5044/13 776) and at the inner-city slum from 23.0% (502/2185) to 32.2% (5514/17 142), but decreased from 15.7% (77/491) to 11.6% (547/4721) at the rural site (Table 5). 

**Table 5:** Change in reporting of illnesses and in average monthly health expenses as a result of a study to determine health diary acceptability, north India, 2012–2019

Parameter	No. (%)							
	Arunachal Pradesh (tribal)			Delhi (slum)			Hardoi (rural)	
	Baseline^a^ (*n* = 2 205)	Diary pages retrieved (*n* = 13 776)		Baseline^a^ (*n* = 2 185)	Diary pages retrieved (*n* = 17 142)		Baseline^a^ (*n* = 491)	Diary pages retrieved (*n* = 4 721)
**Illness reported**								
Yes	399 (18.1)	5 044 (36.6)^b^		502 (23.0)	5 514 (32.2)^b^		77 (15.7)	547 (11.6)
No	1 806 (81.9)	8 732 (63.4)		1 683 (77.0)	11 628 (67.8)		414 (84.3)	4 174 (88.4)
**Monthly health expenses (Indian rupees)^c^**								
Nil	2 116 (96.0)	12 412 (90.1)		2 135 (97.7)	12 926 (75.4)		296 (60.3)	4 429 (93.8)
< 6 298	55 (2.5)	1280 (9.3)		22 (1.0)	3 977 (23.2)		50 (10.2)	170 (3.6)
6 298–10 495	18 (0.8)	28 (0.2)		4 (0.2)	34 (0.2)		16 (3.3)	47 (1.0)
10 496–15 705	8 (0.4)	14 (0.1)		9 (0.4)	17 (0.1)		33 (6.7)	47 (1.0)
15 706–20 991	8 (0.4)	14 (0.1)		15 (0.7)	171 (1.0)		96 (19.6)	28 (0.6)
> 20 991	0 (0.0)	28 (0.2)		0 (0.0)	17 (0.1)		0 (0.0)	0 (0.0)

^a^ As recorded in baseline survey determining sociodemographic properties of those who agreed to take part in health diary project.

^b^ Significant rise (*P* < 0.05) in reporting illnesses using health diary at tribal area and inner-city slum.

^c^ Expenses categorized as per Kuppuswamy scale for 2017.^9^

Note: The 2016–2017 currency rate was one United States dollar to 67 Indian rupees.^10^

We noted a large reduction in health costs in the second-highest expense category in the rural area from 19.6% (96/491) to 0.6% (28/4721), corresponding to an increase in lowest-category expenses from 60.3% (296/491) to 93.8% (4429/4721; Table 5).

We present a morbidity profile of all illnesses reported in the collected diary pages (total no. 11 105) in Table 6. Of all medical issues reported, influenza-like illnesses (fever, cough, headache) were the most prevalent. Our data show that such illnesses were reported with the highest frequency of 58.9% (2972/5044) at the tribal site, followed by 44.4% (243/547) at the rural site and 30.1% (1661/5514) at the inner-city slum. The most frequently reported symptom at the inner-city slum site was that of abnormal involuntary movements (1439/5514; 26.1%), reported at lower levels at the tribal (574/5044; 11.4%) and rural (59/547; 10.8%) sites. We observed that menstrual disorders were more prevalent at the rural (13/547; 2.4%) and inner-city slum (169/5514; 3.1%) sites as compared with the tribal site (28/5044; 0.6%). Our health diary data show that functional intestinal disorders were most frequent at the tribal site (131/5044; 2.6%) compared with the inner-city slum (107/5514; 1.9%) and rural site (2/547; 0.4%). 

**Table 6:** Prevalence of illnesses reported in retrieved diary pages, in study to determine diary acceptability, north India, 2012–2019

Symptom	ICD-10 code	No. (%)		
		Arunachal Pradesh (tribal) (*n* = 5044)	Delhi (slum) (*n* = 5514)	Hardoi (rural) (*n* = 547)
Gastroenteritis and colitis	A9	132 (2.6)	148 (2.7)	16 (2.9)
Mental and behavioural disorders due to alcohol	F10	12 (0.2)	73 (1.3)	17 (3.1)
Visual disturbances	H53	104 (2.1)	124 (2.2)	16 (2.9)
Suppurative and unspecified otitis media	H66	117 (2.3)	34 (0.6)	19 (3.5)
Stroke	I64	0 (0.0)	32 (0.6)	0 (0.0)
Disorders of teeth, diseases of lip and oral mucosa	K08, K13	94 (1.9)	74 (1.3)	1 (0.2)
Dyspepsia, functional intestinal disorders	K30, K59	131 (2.6)	107 (1.9)	2 (0.4)
Diseases of digestive system	K92	78 (1.5)	12 (0.2)	1 (0.2)
Vaginal and menstruation disorders	N89, N92	28 (0.6)	169 (3.1)	13 (2.4)
Pregnancy complications	O22	26 (0.5)	25 (0.5)	0 (0.0)
Cough	R05	1327 (26.3)	359 (6.5)	87 (15.9)
Abnormalities of breathing	R06	11 (0.2)	30 (0.5)	6 (1.1)
Pain in throat and chest	R07	109 (2.2)	365 (6.6)	21 (3.8)
Abdominal and pelvic pain	R10	374 (7.4)	389 (7.1)	40 (7.3)
Nausea and vomiting	R11	46 (0.9)	23 (0.4)	2 (0.4)
Abdominal distension	R14	14 (0.3)	188 (3.4)	25 (4.6)
Disturbances of skin sensation	R20	189 (3.7)	396 (7.2)	52 (9.5)
Rash and non-specific skin eruption, localized swelling, mass and lump of skin	R21, R22, R23	2 (0.0)	39 (0.7)	3 (0.5)
Abnormal involuntary movements, convulsions	R25, R56	574 (11.4)	1439 (26.1)	59 (10.8)
Dizziness, giddiness and lack of coordination	R27	12 (0.2)	112 (2.0)	8 (1.5)
Pain and problems associated with urinating	R30, R35	19 (0.4)	74 (1.3)	3 (0.5)
Fever	R50	776 (15.4)	415 (7.5)	101 (18.5)
Headache, fatigue	R51, R53	869 (17.2)	887 (16.1)	55 (10.1)

ICD-10: International Statistical Classification of Diseases and Related Health Problems.^11^

## Discussion

The main benefit of our study was that, by putting the data collection tool in the custody of the community itself, regular and accurate updating of data records became possible. As well as increased transparency and accountability in health data reporting, long-term benefits regarding earlier diagnoses of illnesses can lead to reduced lifetime health expenses and improved quality of life. In the completion of health diaries, we observed a large variation in the number of diary pages completed from one month to another; reductions in some months may have been the result of poor weather preventing volunteers from travelling or a change in focus towards the end of the year-long completion period. We also recorded an increase in those seeking health care in the tribal and slum areas; this increased awareness of health issues may be the result of regular visits from study staff and health volunteers. Our data also show a reduction in spending on health within the top two expense categories in the rural area, which may be due to the timely referrals of project staff. A longer-duration study may show more conclusive trends.

A recent review of the use of home-based records discovered many other benefits in the form of enhanced communication and engagement for both patients and health workers, despite logistical issues with electronic records, and concluded that home-based records should be considered as an important and useful tool in the context of lower literacy and weak health-care systems.^12^ Another study recently examined the distribution and use of maternal and child health home-based records in the poorest women in the low-income country of Afghanistan,^13^ and found it an efficient tool for reporting illnesses. The World Health Organization (WHO) have also published a practical guide for the use of use of home-based records in immunization programmes, addressing the lack of guidance on the content and design of such records.^14^

Our motivation to record data on influenza-like illnesses and seemingly transient or trivial symptoms such as fever and headaches, which was timely in light of the coronavirus disease 2019 pandemic, was that such knowledge is important in preventive interventions and in managing public health crises. Other health data generated in our study, also previously unrecorded by front-line health workers, included the prevalence of diabetes or cardiovascular diseases, the awareness of which is vital for control measures. The usefulness of our wide-ranging health data was demonstrated in being able to quickly identify 396 potential patients for a newly opened skin clinic in Delhi.

We examined the cost–effectiveness of our health diary project by measuring cost, outcome and sustainability according to WHO guidelines.^15^^,^^16^ The cost of our human resources, infrastructure, and design and publication of the health diaries amounted to 2 million Indian rupees for 2500 people (or 800 Indian rupees per person, equating to 11.9 United States dollars, US$)^10^ for one year. The use of sponsors’ messages on the front and back pages of the diary (e.g. for information, education and communication about preventive health care) may further reduce this cost in the future. According to a WHO cost and sustainability assessment of health care for the year 2007–2008, the cost of health-care delivery is US$ 16.13 per person (i.e. 788 Indian rupees for the year 2008, or 1097 Indian rupees for the year 2016).^17^ The cost of training and system improvements was met by funding already provided under the existing National Health Mission^18^ without any need for an extra budget.

Regarding sustainability, we incorporated all major factors previously mentioned^19^ critical to health system improvements, such as stakeholder involvement at the planning stage and using existing infrastructure. For example, our data identified a demand for cataract operations at the rural site, so the district hospital van was mobilized in order for patients to have their operation within their community. Similarly, at the rural site, our diary data identified a shortage of food, so we connected farmers with appropriate authorities to obtain seeds to grow vegetables in their village. Some community members across all sites included requests for contraception in their diary pages, allowing us to notify relevant authorities and arrange delivery of condoms and intrauterine devices.

The biggest challenge to our study was the reluctance of the existing health system staff to participate: they cited a lack of directives from management and no incentive to increase efficiency. We also met resistance from existing health workers, who considered involvement in the health diary project as additional work. The private health sector was reluctant to share information. We anticipate a reduction in such resistance in view of the recent health policy changes in India in the form of a WHO-recommended health management information system.^20^ The National Digital Health Mission has now proposed individual health identification numbers and a health data collection system, for which our study provided field data and analysis.

We also experienced an initial reluctance to share health data among diary users, but that changed when the benefits in terms of preventive health education, information about available health schemes and referrals became apparent. Compliance improved further as the diary users obtained access to health services (deworming medication and iron and folic acid supplements) and screening tests (blood glucose and blood pressure) as required. 

Our study had several limitations. These included delays in data entry as a result of transcription only taking place once a month, and a lack of internet connection, both contributing to a disruption in the data flow. A data-generating field study in a low-resource region in Zambia met similar issues in establishing an electronic health record system for the community,^21^ highlighting a need for hybrid solutions. Another limitation was that we were dependent on local authorities for identification of suitable households, which led to delays in progress. We were also limited in terms of skilled health workers (meaning we had to rely greatly on volunteers), a lack of infrastructure to address community demand, and the large loss to follow-up as a result of migration and lost or damaged diaries. Previous studies based in both Ghana and India have reported similar challenges.^22^^,^^23^

In the next phase of our study, we aim to introduce and validate robust internet-based applications, reduce out-of-pocket costs for the community by providing a cost-effective continuum of care and further support patients in accessing appropriate health-care information. We also intend to conduct a more detailed analysis of our data for regional and sociodemographic differences.

We observed good levels of acceptability and compliance across all three types of low-income community with our health diary project. From our initial field studies, we have observed how our study participants have benefited from timely preventive education and referrals, while also equipping service providers with data on the health and requirements of the population, allowing improved planning.^24^ Since the majority of our diary users required assistance in recording their symptoms, we have also identified a need for more front-line health workers with the relevant skills. 
